# A novel mode of induction of the humoral innate immune response in *Drosophila* larvae

**DOI:** 10.1242/dmm.027102

**Published:** 2017-03-01

**Authors:** Hiroyuki Kenmoku, Aki Hori, Takayuki Kuraishi, Shoichiro Kurata

**Affiliations:** 1 Department of Molecular Biopharmacy and Genetics, Graduate School of Pharmaceutical Sciences, Tohoku University, Sendai 980-8578, Japan; 2 Graduate School of Medical Sciences, Kanazawa University, Ishikawa 920-1192, Japan; 3 Department of Microbiology and Immunology, Keio University School of Medicine, Tokyo 160-8582, Japan; 4 Faculty of Pharmacy, Institute of Medical, Pharmaceutical and Health Sciences, Kanazawa University, Ishikawa 920-1192, Japan; 5 PRESTO, Japan Science and Technology Agency, Tokyo 102-0076, Japan

**Keywords:** Innate immunity, *Drosophila*, Larvae

## Abstract

*Drosophila* adults have been utilized as a genetically tractable model organism to decipher the molecular mechanisms of humoral innate immune responses. In an effort to promote the utility of *Drosophila* larvae as an additional model system, in this study, we describe a novel aspect of an induction mechanism for innate immunity in these larvae. By using a fine tungsten needle created for manipulating semi-conductor devices, larvae were subjected to septic injury. However, although Toll pathway mutants were susceptible to infection with Gram-positive bacteria as had been shown for *Drosophila* adults, microbe clearance was not affected in the mutants. In addition, *Drosophila* larvae were found to be sensitive to mechanical stimuli with respect to the activation of a sterile humoral response. In particular, pinching with forceps to a degree that might cause minor damage to larval tissues could induce the expression of the antifungal peptide gene *Drosomycin*; notably, this induction was partially independent of the Toll and immune deficiency pathways. We therefore propose that *Drosophila* larvae might serve as a useful model to analyze the infectious and non-infectious inflammation that underlies various inflammatory diseases such as ischemia, atherosclerosis and cancer.

## Abstract (precis)

**Editors' choice:** *Drosophila* larvae represent a useful model of infectious inflammation and sterilely induced humoral innate response, which are relevant for disorders associated with chronic inflammation.

## INTRODUCTION

*Drosophila* adults have been used as a leading model organism to investigate molecular mechanisms of innate immunity (Lemaitre and Hoffmann, 2007; Buchon et al., 2014) since it was first demonstrated in 1996 that the Toll pathway, which was initially characterized as an essential pathway for dorsoventral patterning in *Drosophila* embryos (Anderson et al., 1985a,b), was required for the induction of the antifungal peptide gene *Drosomycin* (*Drs*) upon fungal infection (Lemaitre et al., 1996). In particular, *Drosophila* adult models have contributed to identifying genes required for the humoral innate immune responses and for the production of antimicrobial peptides (AMPs) and melanization factors (Lemaitre et al., 1995; Rämet and Hultmark, 2014). In *Drosophila* adults, AMP induction upon challenge with microbes is controlled by two distinct signaling pathways, the Toll and immune deficiency (IMD) pathways (Lemaitre and Hoffmann, 2007; Valanne et al., 2011; Myllymäki et al., 2014). The Toll pathway is required for the induction of *Drs* and for survival following systemic infection with Gram-positive bacteria or fungi (Ferrandon et al., 2007). Specifically, the recognition of lysine-type peptidoglycans or β-glucans from microbes by the PGRP-SA/GNBP1 complex or by GNBP3 in the hemolymph activates modular serine protease (ModSP), followed by activation of Spätzle (Spz)-processing enzyme and cleavage of Spz, a protein ligand of the Toll receptor (Gottar et al., 2002, 2003, 2006; Jang et al., 2006; [Bibr DMM027102C9][Bibr DMM027102C10]). In addition, so-called ‘danger signals’ also activate the Toll pathway through the protease Persephone (Psh). For example, exogenous danger signals such as PR1 secreted from pathogenic fungi, as well as endogenous danger signals generated in apoptosis-deficient mutants, lead to the activation of Psh and subsequent processing of Spz (Chamy et al., 2008; Ming et al., 2014; Obata et al., 2014). The active form of Spz induces conformational changes in the Toll receptor, activates Toll intracellular signaling (Kanoh et al., 2015b) and ultimately leads to the nuclear translocation of nuclear factor-kappa B (NF-κB) proteins Dif and Dorsal, inducing the expression of antimicrobial peptide genes including *Drs* (Lindsay and Wasserman, 2014). Conversely, the IMD pathway recognizes diaminopimelic acid-type peptidoglycans derived from Gram-negative bacteria via peptidoglycan recognition protein (PGRP)-LC and PGRP-LE (Kleino and Silverman, 2014). These receptors facilitate downstream signaling via the adaptor protein IMD, activate the NF-κB protein Relish, and induce the expression of antimicrobial peptides such as *Diptericin* (*Dpt*) (Paquette et al., 2010). Notably, these pathways are essentially characterized in *Drosophila* adults.

In contrast, *Drosophila* larvae have been largely utilized for dissecting cellular immune responses, particularly for nematode and wasp infections (Paddibhatla et al., 2010; Arefin et al., 2015; Kucerova et al., 2015; Hillyer, 2016). Insect hemocytes, representing blood cells, are composed of three cell types: plasmatocytes, crystal cells, and lamellocytes. These play central roles in cellular immunity by phagocytosing bacteria (plasmatocytes), involvement in the melanization process (crystal cells) and forming capsules around wasp eggs, a process referred to as encapsulation (lamellocytes) (Honti et al., 2014; Gold and Brückner, 2015; Parsons and Foley, 2016). For example, recent studies have begun to unravel the complex encapsulation processes by using *Drosophila* larvae upon infection with parasitoid wasps such as *Leptopilina boulardi* (Kari et al., 2016). In addition, the fat body, an immune-responsive organ in flies functionally resembling the mammalian liver, expresses *edin* and utilizes Toll signaling to control the numbers of plasmatocytes (Schmid et al., 2014; Vanha-aho et al., 2015). Finally, JAK-STAT signaling in somatic muscles is important for inducing the encapsulation reaction and controls the number of circulating lamellocytes (Yang et al., 2015).

By contrast, only a handful of studies have been published related to use of the *Drosophila* larval model of bacterial infection to analyze humoral immune responses (Ferrandon et al., 1998; Manfruelli et al., 1999; Ligoxygakis et al., 2002; Shia et al., 2009; Yamamoto-Hino et al., 2015; Yamamoto-Hino and Goto, 2016). Because these studies implicate intriguing differences in terms of the induction mechanisms of AMPs between larvae and adults, a larval model might thus have the potential to identify novel molecular mechanisms. However, it is possible that the limited numbers of publications on larval bacterial infection might partly be due to technical difficulties in the manufacture of uniform tungsten wires sharpened by electrolysis and their use in introducing infections (Romeo and Lemaitre, 2008) without causing severe damage that leads to the death of the larvae. Consistent with this likelihood, the survival and colony-forming assays upon systemic infection in larvae have been seldom reported. Here, we present a method to perform larval infection using a tungsten needle provided by a manufacturer that produces pins for testing semi-conductor devices. By using this uniform and solid needle, we were able to successfully perform and investigate bacterial infection in *Drosophila* larvae. In addition, we found that mechanical stimuli generated by pinching larvae with forceps resulted in the sterile induction of a antimicrobial peptide, providing a novel model for non-infectious activation of the humoral innate immune response.

## RESULTS

### The Toll pathway is required for survival against Gram-positive bacterial infection in larvae but not for bacterial removal

**Fig. 1.**
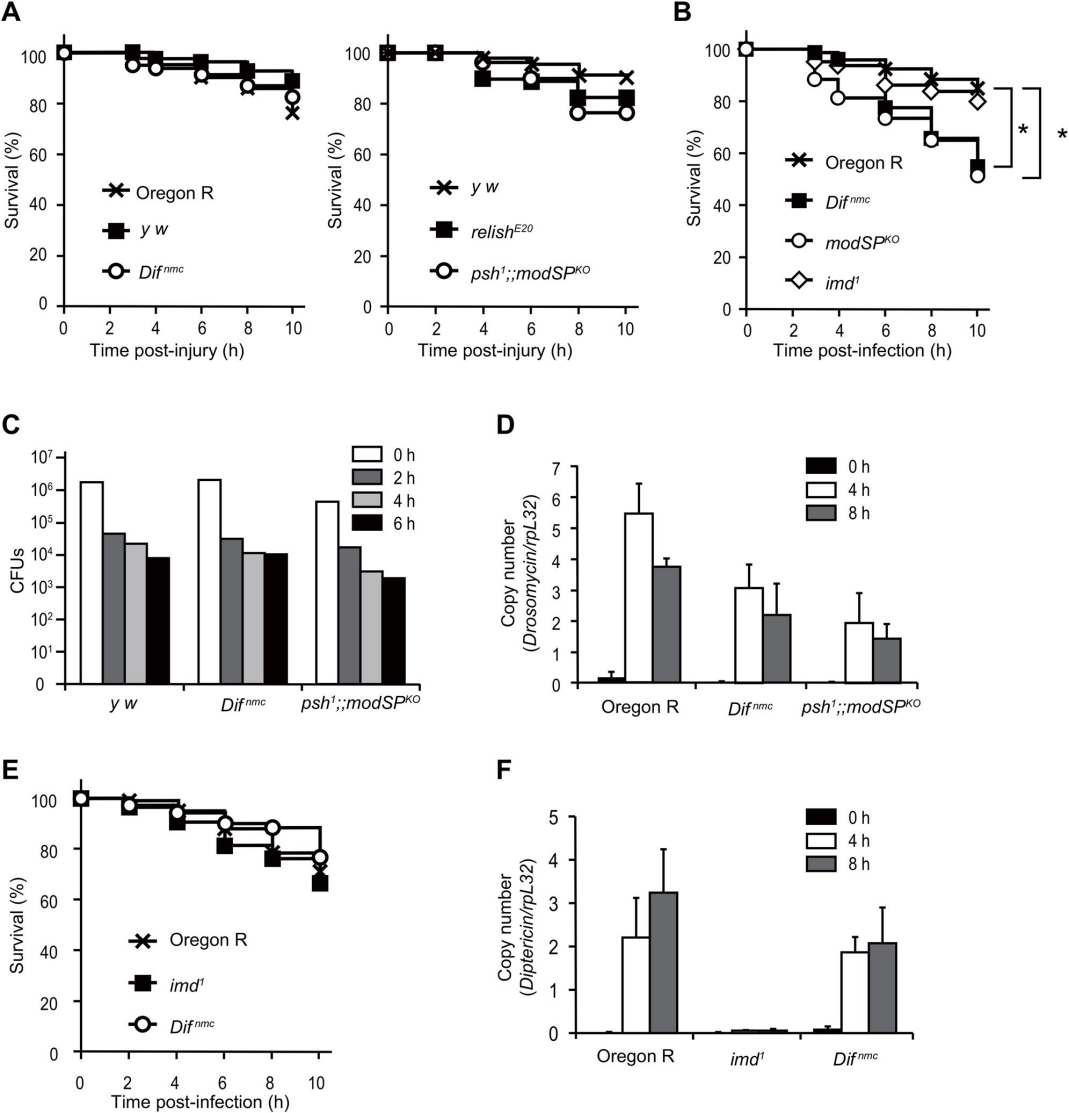
**Systemic infection in *Drosophila* larvae by septic injury with a fine tungsten needle.** (A) Survival analysis of larvae upon clean injury. Oregon R wild-type larvae, wild-type control *y w*, Toll pathway mutant *Dif^nmc^*, *psh* and *modSP^KO^* double mutant, and the IMD pathway mutant *relish^E20^* were used. (B) Survival analysis of larvae upon septic injury with *S. saprophyticus*. Larvae of Oregon R, *Dif^nmc^*, *modSP^KO^* and *imd^1^* mutant were used. (C) Colony forming unit (CFU) assay before (0 h) and after septic injury with *S. saprophyticus* at the indicated time points. Larvae of *y w*, *Dif^nmc^*, and *psh^1^* and *modSP^KO^* double mutant were used. (D) Real-time qPCR analysis of antimicrobial peptide *Drs* expression upon septic injury with *S. saprophyticus* at the indicated time points with larvae of Oregon R, *Dif^nmc^*, and the *psh^1^* and *modSP^KO^* double mutant. (E) Survival analysis of larvae upon septic injury with *Ecc15*. Larvae of Oregon R, *Dif^nmc^*, *modSP^KO^* and *imd^1^* mutants were used. Each survival curve is representative of at least two independent experiments of 60 larvae each (A,B,E). *P*-values were calculated using the log-rank test. (F) Real-time qPCR analysis of antimicrobial peptide *Dpt* expression upon septic injury with *Ecc15* at the indicated time points with larvae of Oregon R, *Dif^nmc^*, and *imd^1^* mutants. Data are representative of more than two independent experiments performed in 20 larvae (C,D,F) (*P<0.05).

To easily and consistently perform infection using third instar larvae, we employed a fine tungsten needle used for the examination of semiconductor devices. With this needle, over 80% of larvae were able to survive following a clean injury in the wild type and in Toll pathway and IMD pathway mutants (Fig. 1A). By pricking larvae with a needle dipped into a pellet of Gram-positive bacteria *Staphylococcus saprophyticus*, we found that Toll pathway mutants were susceptible to the infection (Fig. 1B), although the number of bacteria in the infected whole mutant larvae after any time point was similar to that in the wild type (Fig. 1C). These results suggest that the Toll pathway is dispensable for bacterial clearance in larvae, showing a sharp contrast to the results from *Drosophila* adults in which the Toll pathway is required for the removal of bacteria upon Gram-positive bacterial challenge. Notably, although the induction of the antifungal peptide gene *Drs* was slightly lower in Toll pathway mutants than in wild-type larvae, substantial induction of *Drs* still remained in the mutants (Fig. 1D), consistent with the results of Manfruelli et al. (1999).

We next challenged larvae with Gram-negative bacteria using the needle. Fig. 1E and F show that IMD mutant larvae were not sensitive to infection with *Ecc15*, although the induction of the antibacterial peptide gene *Dpt* was almost abrogated in the mutant. From these results, we conclude that survival, AMP expression and bacterial number upon bacterial infection by septic injury with a tungsten needle could be consistently measured in *Drosophila* larvae, and that the role of the Toll pathway was somewhat different during this process compared with the adult infection model.

### Pinching by forceps induces the expression of AMP in larvae

**Fig. 2.**
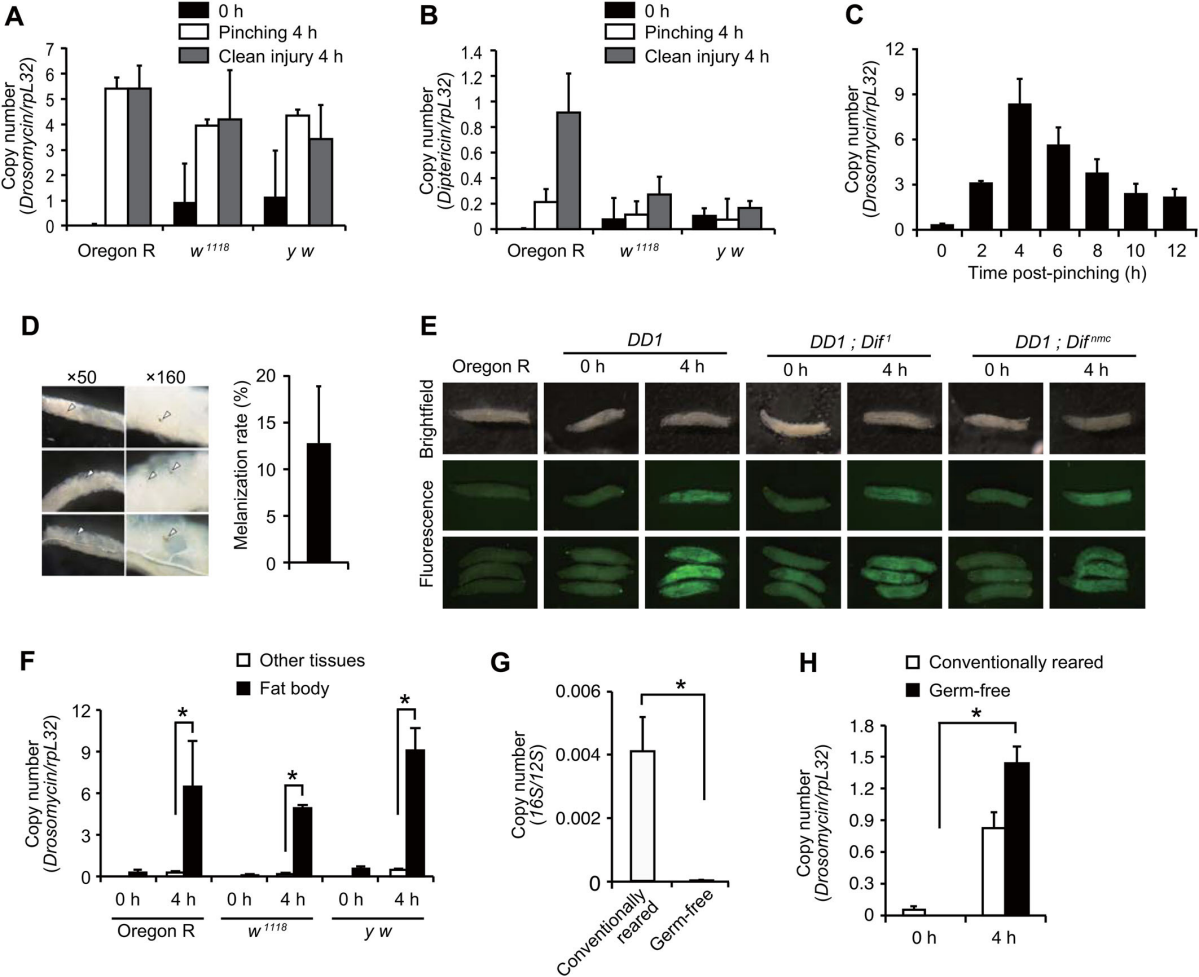
**Characterization of antimicrobial peptide induction following pinching larvae with forceps.** (A-C) Real-time qPCR analysis of *Drs* expression (A,C) or *Dpt* expression (B) upon clean injury or pinching larvae with forceps at the indicated time points with larvae of Oregon R, *w^1118^* and *y w.* Pinching was performed with larvae of *w^1118^* in C. (D) Melanization spots after pinching larvae with forceps after 4 h. The indicated magnification of the objective lens was used; arrowheads indicate melanization spots. The right bar graph shows the percentage of larvae that exhibited melanization spots. Data were analyzed by Student's *t*-test and values represent the means±s.e. of three independent experiments with 30 larvae each. (E) *Drs*-GFP reporter analysis using a fluorescence stereomicroscope upon pinching larvae with forceps after 4 h. Larvae of Oregon R, *Drs-GFP Dpt-lacZ (DD1); c564-GAL4*, *DD1; Dif^1^* and *DD1; Dif^nmc^* were used. Brightfield (top row) and fluorescence images (bottom 2 rows) of single larva or multiple larvae (bottom row); the green signal indicates GFP fluorescence. (F) Real-time qPCR analysis of *Drs* expression following pinching of Oregon R, *w^1118^,* and *y w* larvae with forceps at the indicated time points*.* The larval fat body was dissected out from other tissues. (G,H) Real-time qPCR analysis of bacterial genomic DNA coding for 16S rRNA, which was normalized by the *Drosophila* genomic region for 12S RNA from conventionally reared or germ-free Oregon R larvae before pinching (G), or of *Drs* expression following pinching larvae with forceps at 4 h with conventionally reared or germ-free Oregon R larvae (H). Data were analyzed by the Student's *t*-test and values represent the means±s.e. of three independent experiments with 10 larvae each (F,G). *P<0.05.

We found that clean injury with the needle induced the expression of *Drs* and *Dpt* (Fig. 2A,B). Furthermore, even pinching larvae using forceps, a normal means of handling larvae, caused strong *Drs* induction (Fig. 2A). Time-course experiments showed that *Drs* expression was induced from 2 h, maximized at 4 h and continued to 12 h (Fig. 2C). The level of *Drs* after eclosion was not increased compared with the level in untreated flies (Fig S4A). After pinching, 10% of larvae showed small melanized spots (Fig. 2D), although extremely weakly pinched larvae did not show melanization and the level of *Drs* induction was marginal (Fig. S4B), implying that pinching might cause minor injury in larval tissues. Next, we examined which tissues exhibited *Drs* expression. Fig. 2E shows that *Drs* reporter larvae exhibited GFP signals in the whole fat body and that the position of pinching was not connected with *Drs* induction. Consistent with this result, quantitative real-time-polymerase chain reaction (real-time qPCR) analysis showed that the induction of *Drs* was detected in the fat body dissected out from other tissues (Fig. 2F). These results indicate that *Drs* is induced in the fat body upon pinching with forceps.

As *Drosophila* possess commensal bacteria (Kuraishi et al., 2013), *Drs* induction by pinching might be caused by such infections. To assess this possibility, germ-free larvae (Fig. 2G) were used for pinching experiments. Fig. 2H shows that the level of induction of *Drs* in germ-free larvae was not reduced compared with that in conventionally reared larvae, indicating that *Drs* expression is sterilely induced by pinching with forceps. Next, we performed microarray analysis using pinched larvae in order to examine whether *Drs* was uniquely induced by pinching or whether other defense response genes that respond to infection in adults (De Gregorio et al., 2001, 2002) were also induced. We found that in addition to *Drs*, several immune-related genes such as *IM1*, *IM3*, *IM10* and *Attacin* were induced over 10-fold upon pinching with forceps (Table 1). In addition, stress responsive genes such as *TotA*, *TotB* and *TotC* were induced in the larvae. This result suggests that pinching larvae with forceps induces a humoral innate immune response that is similar to that observed in systemic infection in adults. We also noticed that a number of chitin metabolic genes were also downregulated upon pinching (Table 2).

**Table 1.**
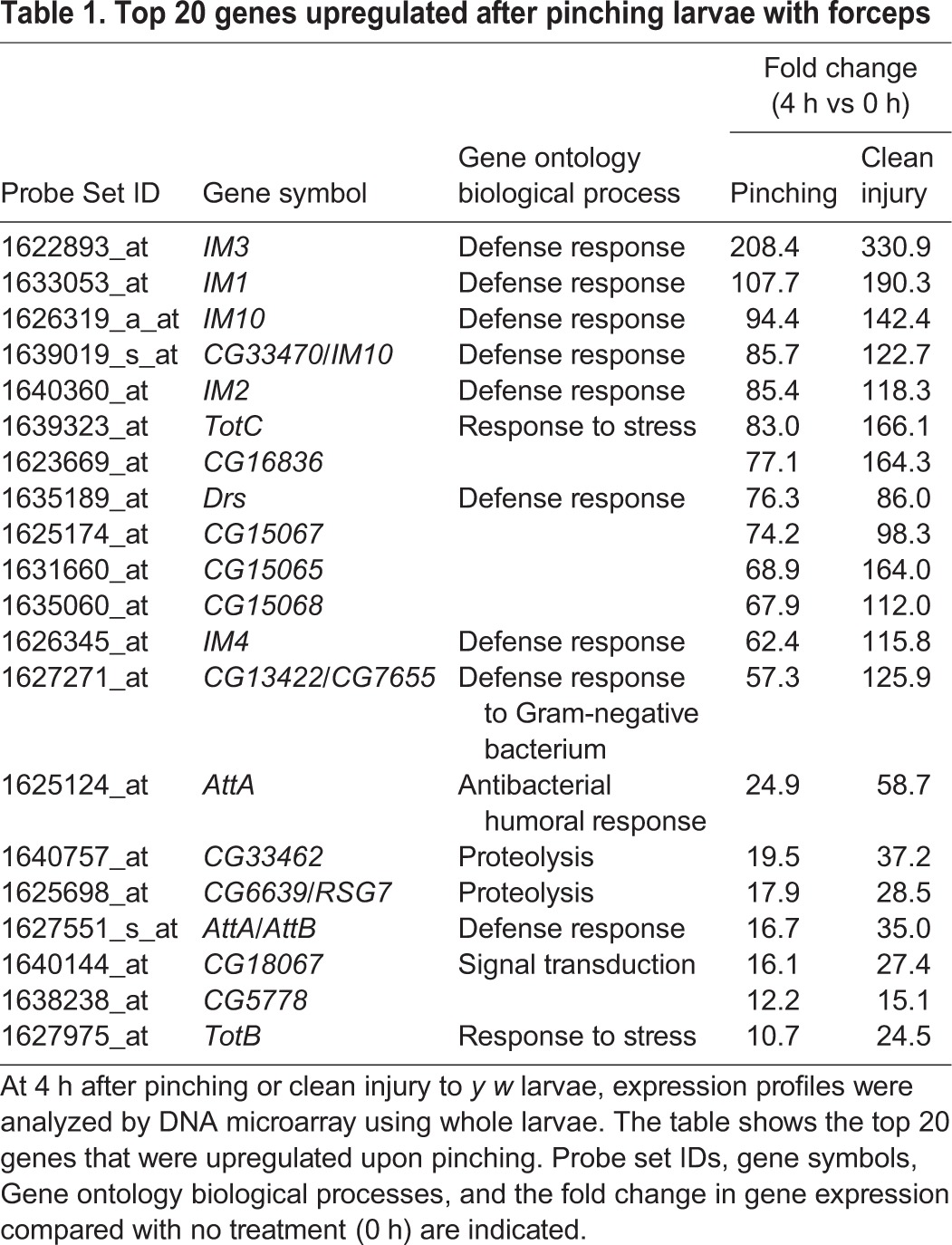
**Top 20 genes upregulated after pinching larvae with forceps**

**Table 2.**
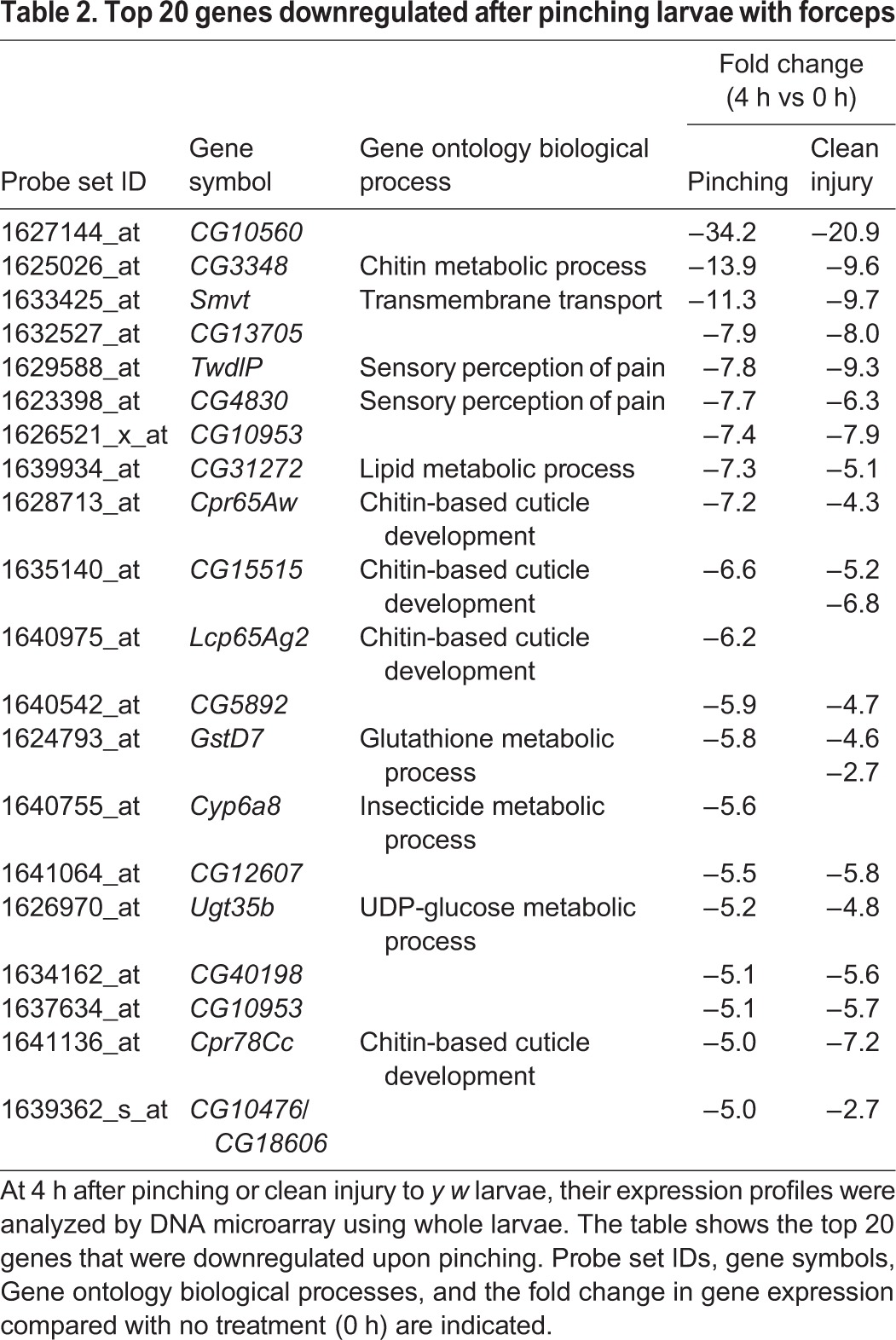
**Top 20 genes downregulated after pinching larvae with forceps**

### Toll pathway genes contribute to the induction of *Drs* upon pinching with forceps

**Fig. 3.**
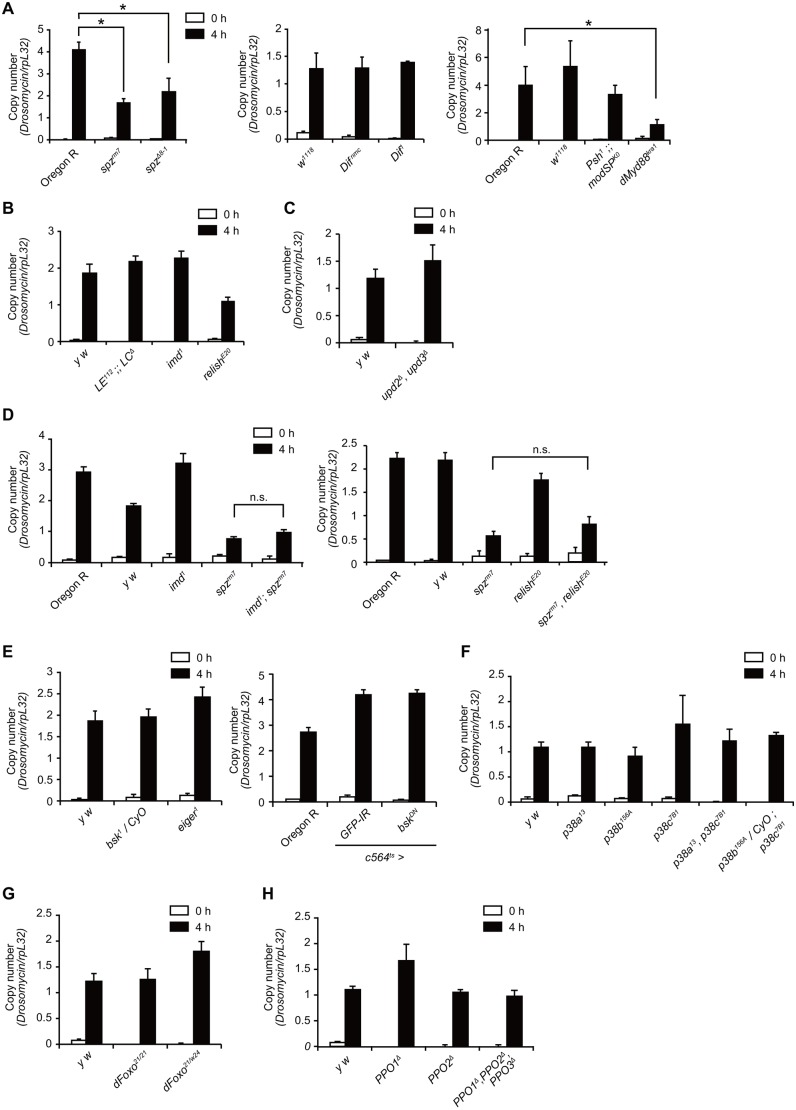
***Drs* induction in several mutant larvae upon pinching with forceps.** (A-H) Real-time qPCR analysis of *Drs* expression after pinching larvae with forceps at 4 h with larvae of Oregon R, *w^1118^*, *spz^rm7^*, *spz^Δ8-1^*, *psh^1^;;modSP^KO^*, *Dif^nmc^*, *Dif^1^* and *dMyd88^kra1^* (A); larvae of *y w*, *pgrp-le* and *pgrp-lc* double mutant (*LE^112^;;LC^Δ^*), *imd^1^*, and *relish^E20^* (B); larvae of *y w* and *upd2^Δ^ upd3^Δ^* (C); larvae of Oregon R, *y w*, *imd^1^*, *spz^rm7^*, *imd^1^;spz^rm7^*, *reslih^E20^*, and *spz^rm7^ relish^E20^* (D); larvae of Oregon R, *y w*, *bsk^1^/CyO*, *eiger^1^*, *GPF-IR,* or *bsk^DN^* driven by *c564-GAL4 tubP-GAL80^ts^* (E); larvae of *y w*, *p38a^13^*, *p38b^156A^*, *p38c^7B1^*, *p38a^13^ p38b^156A^*, and *p38b^156A^/CyO ; p38c^7B1^* (F); larvae of *y w*, *dFoxo^21^*, and *dFoxo^21^/dFoxo^w24^* (G); and larvae of *y w*, *PPO1^Δ^*, *PPO2^Δ^*, and *PPO1^Δ^ PPO2^Δ^ PPO3^Δ^* (H). Data are representative of at least two independent experiments and were analyzed by Student's *t*-test; values represent the means±s.e. of triplicate samples with 10 larvae each. *P<0.05; n.s., not significant.

Next, we asked which signaling pathway is involved in the induction of *Drs* upon larval pinching. Real-time qPCR analysis showed that the level of induction in the *spz* mutant or *dMyd88* mutant was approximately half that of the wild-type larvae (Fig. 3A). In contrast, the induction of *Drs* was comparable to that in the wild-type in the *Dif* mutant, or *psh* and *modSP* double mutants (Fig. 3A). These results suggest that certain Toll pathway components are partly required for the induction of *Drs* upon pinching. We next investigated IMD pathway mutants and found that larvae of the *pgrp-le* and *pgrp-lc* double mutant, *imd* mutant or *relish* mutant exhibited normal *Drs* induction after pinching (Fig. 3B). Furthermore, the level of induction of *Drs* in the double mutant larvae for *imd* and *spz*, or for *relish* and *spz* was almost the same as that in the *spz* single mutant (Fig. 3D), suggesting that the Toll and IMD pathways did not have a redundant role in pinching-induced *Drs* expression. We further investigated the involvement of the JAK-STAT, JNK, p38, dFOXO and pro-PO pathways, all of which suggested a role for AMP induction or host defense under certain conditions (Kim et al., 2002; Buchon et al., 2009a; Becker et al., 2010; Chen et al., 2010; Binggeli et al., 2014; Parisi et al., 2014). Inhibition of the JAK-STAT pathway by using an *upd2* and *upd3* double mutant did not reduce the induction of *Drs* in pinched larvae (Fig. 3C), implying that the JAK-STAT pathway may be dispensable for *Drs* induction. Similarly, the normal *Drs* induction observed upon pinching in larvae with an *eiger* mutation or *c564-GAL4*-driven expression of a dominant negative form of Bsk implied that there was no requirement of the JNK pathway in *Drs* induction (Fig. 3E). The level of *Drs* induction was also same in wild-type larvae as in the larvae of *p38a*, *p38b* and *p38c* (Fig. 3F), *dfoxo* (Fig. 3G) and *PPO* (Fig. 3H) mutants, indicating that the p38, dFOXO and pro-PO pathways played no role in the induction of *Drs* following larval pinching with forceps.

### Sensory neurons and hemocytes are dispensable for the induction of *Drs* upon pinching larvae with forceps

**Fig. 4.**
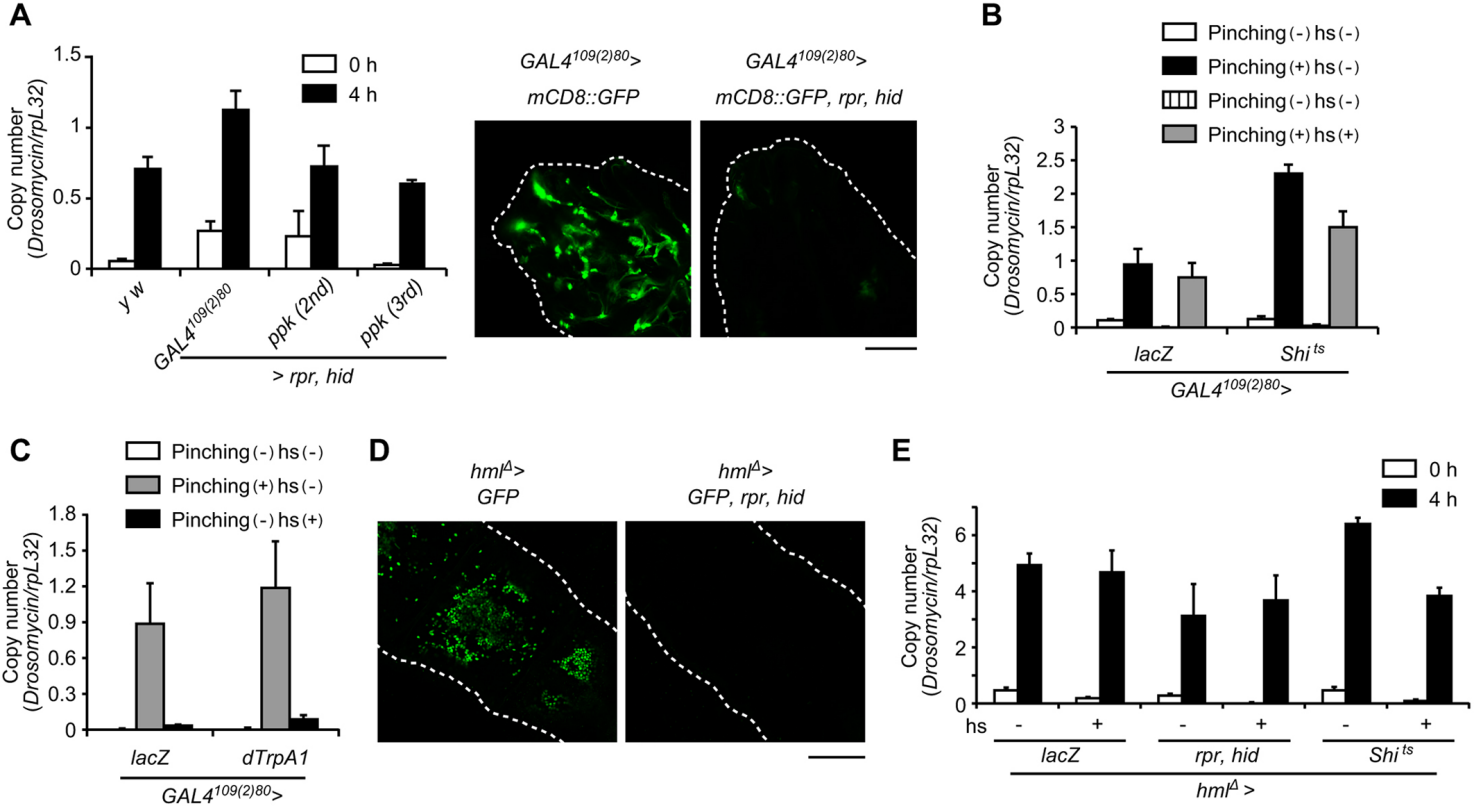
**Sensory neurons and hemocytes are dispensable for the induction of *Drs* upon pinching.** (A) Fluorescence microscopy (right) of larval sensory neurons around the mouth hook. Larvae of *+/+; GAL4109(2)80 UAS-mCD8::GFP/+* and *UAS-rpr UAS-hid/+; GAL4109(2)80 UAS-mCD8::GFP/+* were used. Green indicates the GFP signal and white dotted lines indicate the outlines of each larva. Scale bar: 100 µm. (A-C) Real-time qPCR analysis of *Drs* expression after pinching larvae with forceps for larvae of *y w*, *UAS-rpr UAS-hid ; GAL4^109(2)80^ UAS-mCD8::GFP/+*, *UAS-rpr UAS-hid; ppk-GAL4/+* (second instar stage), *UAS-rpr UAS-hid ;; ppk-GAL4/+* (third instar stage) at 0 or 4 h (A, left); larvae of *UAS-lacZ/GAL4^109(2)80^ UAS-mCD8::GFP, UAS-Shi^ts^/GAL4^109(2)80^ UAS-mCD8::GFP*, with or without pinching and with or without heat shock at 30°C for 4 h (hs) (B); larvae 4 h after treatment in *UAS-lacZ/GAL4^109(2)80^ UAS-mCD8::GFP, UAS-Shi^ts^/GAL4^109(2)80^ UAS-mCD8::GFP*, with or without pinching or heat shock treatment at 37°C for 2 min (hs) (C). Data are representative of at least two independent experiments and were analyzed by the Student's *t*-test; values represent the means±s.e. of triplicate samples with 10 larvae each. (D) Fluorescence microscopy observation of larval hemocytes. Larvae of *hmlΔ-GAL4 UAS-2×EGFP/UAS-lacZ*, and *UAS-rpr UAS-hid/+; hmlΔ-GAL4 UAS-2×EGFP/+* were used. Green indicates the GFP signal and white dotted lines indicate the outlines of each larva. Scale bar: 200 µm. (E) Real-time qPCR analysis of *Drs* expression at 4 h after pinching larvae with forceps of *hmlΔ-GAL4 UAS-2×EGFP/UAS-lacZ*, *UAS-rpr UAS-hid/+; hmlΔ-GAL4 UAS-2×EGFP/+*, *hmlΔ-GAL4 UAS-2×EGFP/+; UAS-Shi^ts^/+*, with or without pinching or treatment at 29°C for 2 days (hs). Data are representative of at least two independent experiments and were analyzed by the Student's *t*-test; values represent the means±s.e. of triplicate samples with 10 larvae each.

When pinching larvae with forceps, we touched the larval cuticle under which the web of sensory neurons exists, prompting us to examine the role of sensory neurons in pinching-induced *Drs* expression. We first ablated sensory neurons by expressing the apoptosis-inducing genes *reaper* and *hid* with a pan-sensory neuron *GAL4^109(2)80^* driver (Fig. 4A, Fig. S5) or class IV sensory neuron *ppk-GAL4* driver. Fig. 4A shows that the level of induction of *Drs* in larvae with sensory neurons ablated by either driver was the same as that in the wild type. We next inhibited the function of sensory neurons with the same drivers by expressing temperature-sensitive Shibire (Kitamoto, 2001). However, no effect was again observed on *Drs* induction when neurotransmission was suppressed (Fig. 4B). Conversely, we then monitored *Drs* expression using larvae in which the sensory neurons were artificially activated by expressing the dTrpA1 ion channel (Hamada et al., 2008). Fig. 4C shows that *Drs* was not induced in the activated larvae without pinching. These results collectively suggest that sensory neurons are not involved in the induction of *Drs* upon larval pinching with forceps.

As Spz has been suggested to be secreted from hemocytes (Shia et al., 2009), we tested the role of hemocytes in the induction of *Drs* upon pinching. We observed that hemocyte-specific expression of *reaper* and *hid* mediated by using a *hmlΔ-GAL4* driver effectively ablated hemocytes in larvae (Fig. 4D). Using these larvae, we next examined the induction of *Drs* upon pinching and found that no difference could be detected between wild-type and hemocyte-ablated larvae with respect to the level of *Drs* induction (Fig. 4E). Consistent with this, inhibition of the phagocytic function of hemocytes by expressing Shibire (Awasaki and Ito, 2004) had no effect on the induction of *Drs* upon pinching (Fig. 4E). These results suggest that hemocytes are dispensable for the induction of *Drs* after pinching with forceps.

## DISCUSSION

In this study, we present a method by which systemic bacterial infection can be performed easily and consistently in *Drosophila* larvae, thus providing another genetically tractable model to decipher infectious diseases. In this model, we show that the role of the Toll pathway in resistance against systemic infection in *Drosophila* larvae differs from that in adults to a certain extent. Specifically, the Toll pathway is likely to be required for tolerance (Ayres and Schneider, 2012) but not for resistance against Gram-positive bacteria, and exhibits partial involvement in the induction of AMPs; the latter being consistent with suggestions from a previous study (Manfruelli et al., 1999). *Drs*, an antimicrobial peptide whose expression is under the control of the Toll pathway, is strongly induced upon Gram-positive bacterial infection, although *Drs* is only active against fungi but not bacteria (Fehlbaum et al., 1994). One possibility to explain the former discrepancy might be that certain genes induced upon infection, including *Drs*, might function in conjunction to confer resistance and tolerance to adults and larvae, albeit with as-yet unknown mechanisms. This point should be elucidated in future research.

While performing these infection studies, we serendipitously found that the humoral innate immune response is activated in *Drosophila* larvae by modest mechanical stimuli; i.e. by pinching larvae with forceps, as they are commonly handled. AMP expression induced by pinching in larvae is sterile and partially independent from known innate immune signaling; these conclusions are supported by the following evidence: (1) the induction of *Drs* was observed in germ-free larvae upon pinching; (2) substantial induction of *Drs* remained in double mutants for the Toll and IMD pathways; (3) normal induction of *Drs* occurred after pinching in p38, JNK, JAK-STAT, dFOXO and pro-PO pathway mutants; and (4) hemocytes were dispensable for the induction of *Drs* upon pinching. These observations support the assertion that *Drosophila* larvae possess a novel mode of induction of the humoral innate immune response that might represent a good model for studying the mechanisms underlying sterile inflammation. Although we demonstrated that Dif, a *Drosophila* NF-κB essential for the induction of *Drs* upon systemic infection in adults, was not involved in pinching-induced *Drs* expression in larvae, we could not rule out the possible involvement of NF-κB in transactivating *Drs* expression, as we were unable to examine the redundant role of the other NF-κB proteins, Dorsal and Relish, because of the unavailability of viable lines. The dependency on NF-κB remains a question to be solved in future studies. Furthermore, we were unable to identify the essential genes required for the induction of *Drs* upon pinching. Unbiased genetic screening might therefore be necessary to unravel the molecular mechanism underlying this phenomenon.

Sterile inflammation is believed to contribute to many pathological conditions such as chronic inflammatory diseases including cancer (Rock et al., 2010). In *Drosophila*, several studies have established a sterile inflammation model in larvae (Shaukat et al., 2015). Ming et al. (2014) established a larval model for sterile AMP induction using a caspase mutant. They showed that the induction of *Drs* is solely dependent on Spz and Persephone, suggesting that the molecular mechanism of *Drs* induction in this mutant is different from our ‘pinching’ model. In addition, Hauling et al. (2014) and Parisi et al. (2014) reported that tumors can induce the expression of AMPs. Parisi et al. (2014) demonstrated that the induction of *Drs* is dependent on *eiger* and *spz*, both of which are not essential for our pinching-induced expression of *Drs*. Furthermore, Kanoh et al. (2015a) showed that *Drosophila* larvae possess another intrinsic ligand for the Toll receptor in addition to Spz, although its molecular nature has not yet been identified. Together, these reports suggest that *Drosophila* larvae possess multiple modes of induction of AMPs in response to various sterile stimuli that activate innate immunity.

In the current study, we showed that pinching stimuli can induce AMP expression; however, the physiological relevance of this phenomenon has not yet been elucidated. The larvae of *Drosophila melanogaster* in the wild are expected to be exposed to serious likelihood of attack by parasitoid wasps. Thus, mechanical stimuli might be considered as a potential infectious danger, suggesting that even small injuries resulting from oviposition might be able to activate AMP expression. Consistent with this, Schmid et al. (2014) recently showed that overactivation of Toll signaling could provoke a cellular immune defense that has potential importance in the response to wasp infection.

In conclusion, we demonstrate in this study that *Drosophila* larvae represent a suitable model in which to perform microbial infection by using a fine and uniform tungsten needle and to assess sterile induction of the humoral immune response by pinching larvae with forceps. In particular, because pinching-induced AMP expression is likely to be dependent on an as-yet uncharacterized molecular mechanism, our model might be useful to decipher the complex mechanisms that regulate sterile inflammation, which has considerable importance for the treatment of inflammatory diseases in humans such as ischemia, atherosclerosis and cancer.

## MATERIALS AND METHODS

### Fly stocks and maintenance

*Drosophila* stocks were maintained in standard corn meal-yeast agar medium vials at 25°C. Oregon R, *w^1118^*, and *y w* flies were used as wild-type controls. As Toll pathway mutants, *spz^rm7^* (Morisato and Anderson, 1994), *spz^Δ8-1^* (described below), *modSP^KO^*, *psh^1^;;modSP^KO^* (a gift from Dr Bruno Lemaitre) (Chamy et al., 2008; Buchon et al., 2009b), *for^R^ Dif^nmc^* (described below), *Dif^1^* (a gift from Drs Jean-Marc Reichhart and Dominique Ferrandon) (Rutschmann et al., 2000) and *dMyd88[kra1]* (a gift from Dr Jean-Luc Imler) (Charatsi et al., 2003) were used. As IMD pathway mutants, *PGRP-LE^112^;;PGRP-LC^E12^* (Gao et al., 1999; Takehana et al., 2004), *imd^1^* (a gift from Dr. Bruno Lemaitre) and *Relish^E20^* (Lemaitre et al., 1995; Hedengren et al., 1999) were used. The following mutants were used: *bsk^1^* [Bloomington *Drosophila* Stock Center (BDSC), 3088], *eiger^1^* (a gift from Dr Masayuki Miura) (Igaki et al., 2002), *upd2^Δ^ upd3^Δ^* (a gift from Dr Bruno Lemaitre) (Osman et al., 2013), *PPO1^Δ^ PPO2^Δ^ PPO3^Δ^* (a gift from Dr Bruno Lemaitre) (Binggeli et al., 2014; Dudzic et al., 2015), *dFoxo^21^* and *dFoxo^w24^* (a gift from Dr Marc Tatar) (Jünger et al., 2003; Weber et al., 2005), as well as *p38^13^*, *p38b^156A^* and *p38c^7B1^* (a gift from Dr Bruno Lemaitre) (Davis et al., 2008; Chen et al., 2010; Chakrabarti, et al., 2014). The following transgenic flies were used: *Drs-GFP Dpt-lacZ* (a gift from Dr Dominique Ferrandon) (Jung et al., 2001), c*564-GAL4*, *GAL4^109(2)80^ UAS-mCD8::GFP* (a gift from Dr Tadashi Uemura) (Gao et al., 1999), *ppk-GAL4* on 2nd instar stage (BDSC, 32078), *ppk-GAL4* on 3rd instar stage (BDSC, 32079), *hmlΔ-GAL4 UAS-2×EGFP* (Sinenko and Mathey-Prevot, 2004), *UAS-lacZ* (a gift from Dr Manabu Ote), *UAS-shi^ts^* (a gift from Dr Takeshi Awasaki) (Kitamoto, 2001), *UAS-rpr UAS-hid* (a gift from Dr Shigeo Hayashi) (Zhou et al., 1997), *UAS-dTrpA1* (a gift from Dr Paul Garrity) (Hamada et al., 2008), *tubP-GAL80^ts^* (BDSC), *UAS-GFP-IR* (BDSC, 9330), and *UAS-bsk^DN^* (BDSC, 6409).

#### *Dif^nmc^* allele

When using an allele of the *foraging* (*for*) gene, *for^R^* (a gift from Dr Shireen-Anne Davies) (Osborne et al., 1997) we happened to find that the homozygous flies of *for^R^* were susceptible to systemic infection with *S. saprophyticus*. However, trans-heterozygotes of the deficiency line that covers the *for* locus, *for^R^/Df(2L)ED243*, were not sensitive to the infection (Fig. S1A). Consistent with this, *Drs* induction upon *S. saprophyticus* in adults of *for^R^* homozygotes were severely impaired, although *for^R^/Df(2L)ED243* trans-heterozygotes were perfectly normal (Fig. S1B). These results suggest that our *for^R^* line contains a second point mutation that is involved in the Toll pathway. Therefore, we named this mutation ‘nmc’ (natural immunity-mediating component) and performed mapping with several deficiency lines on the second chromosome. We found that deficiencies that covered the *Dif* and *Dorsal* locus, such as *Df(2L)ED1161* or *Df(J4)*, could not recuperate the immune phenotype of *for^R^* (Fig. S1C). Because *Dorsal* is reported not to be required for *Drs* induction in adults upon infection, we investigated the *Dif* locus by PCR. We found that our *for^R^* line contains foreign sequence of approximately 5 kb, possibly representing an accidental transposable element insertion at the third exon of Dif-RC (Fig. S1D and E), using several primers as shown in Fig. S1D or as follows: Dif-genome-PCR-F1-6: 5′-CGT ATC CAC TCC ACC AG-3′; Dif-genome-PCR-F1-7: 5′-ATT GGA AGG GTA GAC ACA TT-3′; Dif-genome-PCR-F1-8: 5′-GGT AGG ACT ACA GCC GTT TA-3′; Dif-genome-PCR-F1-9: 5′-ATG CCA AAC CCT TCC-3′; Dif-genome-PCR-F1-10: 5′-AGA TTG CCG ACC TTA AGA C-3′; Dif-genome-PCR-F1-11: 5′-GAA GGA GGT TGA ATC TCG-3′; Dif-genome-PCR-F1-12: 5′-TTC ATG GGT TCA TCT CAG T-3′; and Dif-genome-PCR-R1-6: 5′-AGC CTA AGC TCC AAT AGA ACT-3′. These results indicate that nmc represents a serendipitous mutation of the *Dif* gene. As the induction levels of *Drs* upon systemic infection with *S. saprophyticus* in *for^R^* hemocytes and *for^R^/Df(2L)ED1161* are comparable (Fig. 1C), the *Dif^nmc^* allele might be considered as a null or strong hypomorphic allele. We used this *for^R^ Dif^nmc^* line as the *Dif* mutant line in this study (note that *for^R^ Dif^nmc^* is denoted *Dif^nmc^* in this manuscript).

#### *spz^Δ8-1^* and *Drs^Δ7-17^* mutants

*spz* or *Drs* mutants were generated using the CRISPR/Cas9 system as described in Kondo and Ueda (2013). A double-gRNA vector was constructed using pBFv-U6.2, pBFv-U6.2B, and the following primers: spz_in1_1_F: 5′-CTT CGT GCT TGT CTT AAG AAG ACA-3′; spz_in1_1_R: 5′-AAA CTG TCT TCT TAA GAC AAG CAC-3′; spz_ex1_1_F: 5′-CTT CGC AGG TGA TTG GCG GAT CGG-3′; spz_ex1_1_R: 5′-AAA CCC GAT CCG CCA ATC ACC TGC-3′; Drs-int1-1-F; 5′-CTT CGA AAA GGT TCT CAC GGA GCT-3′; Drs-int1-1-R: 5′-AAA CAG CTC CGT GAG AAC CTT TTC-3′; Drs-ex1-1-F: 5′-CTT CGC AGC CCC AGT CTG AAG TGC-3′; Drs-ex1-1-R: 5′-AAA CGC ACT TCA GAC TGG GGC TGC-3′. The constructed vector was used to generate the *U6-spz-gRNA* line, by using the *y^1^ w^67c23^; P{CaryP}attP2* (BDSC, 8622) line (performed in BestGene). The *U6-spz-gRNA* line was crossed to *nos-Cas9* (National Institute of Genetics, CAS-0001) as described in Kondo (2014) to generate candidate deletion lines. Genomic DNAs of each candidate mutant were screened by PCR to check for the deletion (Fig. S2) using the following primes: spz-Fw: 5′-GGA ACT GCT AGA ACA ACT ATG GA-3′; spz-Rv: 5′-CAG TAA CAC CAG CTA CCA GT-3′; Drs-Fw: 5′-GTG ACT GCA CAT GTA TCA TCA TAA TTT G-3′; and Drs-Rv: 5′-GTA GGT CGG GAA CAT TAG GG-3′. One line, *spz^Δ8-1^*, was found to harbor a 350 bp deletion that includes the start codon (Fig. S2); in addition, the line, *Drs^Δ7-17^* was found to have a 210 bp deletion that includes the start codon (Fig. S2); thus, we used these lines as *spz* or *Drs* null mutants, respectively.

To provoke neural activity, *ppk-GAL4* or *GAL4^109(2)80^ UAS-mCD8::GFP* was crossed to *UAS-dTrpA1* and maintained at 18°C until they had developed into third instar larvae. In *ppk-GAL4*, third instar larvae were incubated at 29°C prior to assessment. In *GAL4^109(2)80^*, third instar larvae were incubated in a water bath at 37°C twice for 2 min (10 min intervals at 25°C), then maintained at 25°C for 4 h and used for assays.

To inhibit neural activity, *GAL4^109(2)80^ UAS-mCD8: GFP* was crossed to *UAS-shi^ts^* and maintained at 18°C. Third instar larvae were incubated in a water bath at 32°C for 5 min and experiments were performed at 30°C with warmed equipment. Pinched larvae were moved to agar plates and maintained at 25°C.

To remove sensory neurons or hemocytes, *ppk-GAL4*, *GAL4^109(2)80^ UAS-mCD8::GFP*, or *hmlΔ-GAL4 UAS-2×EGFP* were crossed to *UAS-rpr UAS-hid* and maintained at 18°C. Third instar larvae were incubated at 29°C for two days to induce apoptosis and observed under a stereo fluorescence microscope (M205FA, Leica, Wetzlar, Germany) to check the decrease of GFP signal, or used for the assays.

To inhibit exocytosis and phagocytic activity of hemocytes, *hmlΔ-GAL4 UAS-2×EGFP* was crossed to *UAS-shi^ts^* and maintained at 18°C. Third instar larvae were incubated at 29°C for two days, the experiments were performed at 25°C, and the pinched larvae were soon moved to agar plates and maintained at 29°C.

### Microbial infection and pinching

The following pathogens were used for infection: *Ecc15* (IFO3830) and *S. saprophyticus* (GTC0205). For larval infection, overnight bacterial cultures were concentrated by centrifugation, the pellet was washed with phosphate-buffered saline (PBS), and the larvae were then placed on a cold agar plate and pricked with a fine tungsten needle until complete penetration was achieved (Seimi, Sendai, Japan; total length: 43.5 mm; diameter: 0.2 mm; taper length: 2.5 mm) (Fig. S3A) that had been dipped in a pellet of concentrated bacteria (Fig. S3B) and moved to sealed Petri dishes containing apple juice agar. The needle was frequently changed before it became dull.

To monitor survival, 60 larvae of each genotype were incubated at 29°C after infection and the surviving larvae were every 2 h during transfer to fresh apple juice plates.

To assess the bacterial load in larvae, a colony-forming unit (CFU) assay was performed. Larvae were collected and their surfaces were sterilized with 70% ethanol. At total of 20 larvae of each genotype were homogenized in 500 μl nutrient broth (NB) bacterial medium, serially diluted, and plated onto NB medium plates.

For pinching larvae, MilliQ water was poured into *Drosophila* vials and the water and larvae were moved to Petri dishes. The middle part of third instar larvae were gently (0.2-0.25 MPa, Prescale, Fujifilm, Tokyo, Japan) pinched by forceps (Dumont, 0108-5-PO) for about 1 s (Fig. S3C), and then the larvae were moved to sealed Petri dishes containing apple juice agar. Melanization spots and GFP signals in larvae after pinching were observed using a fluorescent stereo microscope.

### Rearing the axenic fly line

To obtain germ-free larvae, embryos were washed with bleach as described in Broderick et al. (2014). Briefly, embryos were rinsed in 70% ethanol for 1 min, placed in a 2.5% solution of sodium hypochlorite for 2 min, and then washed with 70% ethanol for 2 min. Embryos were then rinsed in sterile MilliQ water. Embryos were transferred to sterile foods and developed to larvae.

To check the axenic state, bacterial DNA was extracted from whole larvae and assessed by real-time qPCR using 16S rRNA primers (Suau et al., 1999).

### Total RNA isolation, real-time qPCR, and microarray analysis

Larvae infected with bacteria or pinched by forceps were collected. Total RNA (1 µg), isolated from around 10 larvae using TRIzol reagent (Thermo Fisher Scientific, Waltham, MA, USA), was used for cDNA synthesis with ReverTra Ace reverse transcriptase (Toyobo Ltd., Osaka, Japan) and oligo (dT) 15 primers (Promega, Madison, WI, USA). Using first-strand cDNA (0.5 μl), real-time qPCR was performed using a LightCycler (Roche Diagnostics, Roswell, GA, USA). *rpL32* was used as the internal control. The following primers were used for real-time qPCR (F=forward, R=reverse): *rpL32*: 5′-AGA TCG TGA AGA AGC GCA CCA AG-3′ (F), 5′-CAC CAG GAA CTT CTT GAA TCC GG-3′ (R); *Drs*: 5′-TTG TTC GCC CTC TTC GCT GTC CT-3′ (F), 5′-GCA TCC TTC GCA CCA GCA CTT CA-3′ (R); *Dpt*: 5′-GTT CAC CAT TGC CGT CGC CTT AC-3′ (F), 5′-CCA AGT GCT GTC CAT ATC CTC C-3′ (R); 12S rRNA: 5'-TGG CGG TAT TTT AGT CTA TCT AGA GG-3′ (F), 5′-TAA GCT ACA CCT TGA TCT GA-3′ (R); and 16S rRNA: 5′-CAG GAT TAG ATA CGG TGG TAG T-3′ (F), 5′-TAA CCA CAT GCT CCA CCG CTT-3′ (R).

For microarray analysis, total RNA from *Drosophila* larvae homogenized in TRIzol was isolated using an RNeasy kit (Qiagen, Venlo, The Netherlands). The RNA quality was checked using an Agilent Bioanalyzer 2100 (Agilent Technologies, Santa Clara, CA, USA). Total RNA (1 μg) was amplified and labeled as complementary RNA (cRNA) using an IVT Labeling Kit (Affymetrix, Santa Clara, CA, USA). Affymetrix *Drosophila* Genome 2.0 arrays were hybridized with 30 μg labeled cRNA, washed, stained and scanned (Goto et al., 2010). Data were analyzed by R software (https://www.r-project.org/).

### Statistical analysis

Statistical analyses were performed using the Student's *t*-test or log-rank test, and *P*<0.05 was considered significant.
